# A Novel Round Insulated Tip Papillotome as an Alternative to the Classic Needle-Knife for Precut Sphincterotomy in Endoscopic Retrograde Cholangiopancreatography

**DOI:** 10.1155/2015/972041

**Published:** 2015-08-09

**Authors:** Birol Baysal, Hakan Akin, Omar Masri, Ali Tüzün İnce, Hakan Senturk

**Affiliations:** ^1^Department of Gastroenterology, Bezmialem Vakif University, Faculty of Medicine, 34093 Istanbul, Turkey; ^2^Department of Gastroenterology, Marmara University, Faculty of Medicine, 34899 Istanbul, Turkey

## Abstract

*Objective*. The purpose of this study was to investigate the efficacy and safety of a new round insulated tip papillotome (r-ITP) as compared to that of the classic needle-knife sphincterotome (NKS) in difficult-to-cannulate endoscopic retrograde cholangiopancreatography (ERCP) patients. *Materials and Methods*. Patients with no exclusion criteria and an intact papilla referred for ERCP were invited to participate in the study. “Difficult-to-cannulate” patients, defined as failure to achieve deep biliary cannulation within five minutes from the first touch of papilla, with no more than ten attempts permitted, were randomly assigned for precut sphincterotomy using either the classic NKS or r-ITP. *Results*. Seventy and 69 patients were randomly assigned to the NKS and r-ITP groups, respectively. The groups were comparable regarding age, sex, indications, and associated conditions. There was no statistically significant difference in terms of successful cannulation or post-ERCP complications between the two groups. Only five patients (3.6%) developed mild to moderate post-ERCP pancreatitis and two had mild bleeding. No perforations or deaths were encountered. *Conclusions*. Although the round insulated tip papillotome was not shown to be superior to the classic NKS concerning efficacy and safety when used by an experienced endoscopist, it remains a simple, safe, and efficacious alternative.

## 1. Introduction

Endoscopic retrograde cholangiopancreatography is currently the procedure of choice in the management of several pancreaticobiliary diseases. Being the prerequisite technical step, successful ductal cannulation might be considered as the most important step for the procedure. Success depends on several factors including the underlying indication, patient anatomy, level of experience of the endoscopist, and the availability of specialized accessory catheters. However, even in most expert hands, this might be challenging in some cases, often requiring alternative approaches that significantly increase the time and complications of the procedure. Failure of deep biliary cannulation when using the standard techniques has been reported as 5–15% [1]. Available options to improve the success rate include precut sphincterotomy techniques, physician-controlled guidewires, or referral for more advanced interventions like the rendez-vous or hepatogastrostomy procedures.

**Figure 1:**
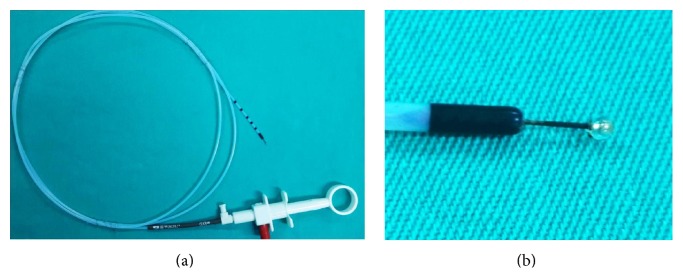
(a) The round insulated tip papillotome used in the study. (b) Note the small porcelain tip in the figure.

Needle-knife sphincterotomy as a mode of precut papillotomy was first introduced by Siegel in 1980 [2]. Several subsequent trials have shown that this technique significantly improves the success rates in difficult-to-cannulate patients. Concerns regarding increased risks of complications, particularly post-ERCP pancreatitis (PEP), were raised in many earlier studies guiding endoscopists to use it only as a last resort or as a rescue technique when all other options failed [3]. However, other confounding factors might have played a role in these observations, like the prolonged procedure time, the excessive manipulation causing ampullary edema, and repeated accidental pancreatic duct cannulation prior to the precut itself, all of which have been shown to be independently associated with increased risks of PEP and other complications [4]. This has led to the new concept of advocating the early use of NKS in difficult-to-cannulate patients [5–7]. The classical NKS is technically simple to use. However, it was postulated that the bare metallic tip of the needle may conduct the electrical current to the pancreatic duct resulting in injury and edema, and thus increasing the risk of PEP [4]. Moreover, this sharp needle tip might theoretically increase the risk of bleeding and perforation. Trying to overcome these potential drawbacks of NKS and to improve the cannulation success rate, Park et al. conducted a noncomparative pilot study [8] using a novel papillotome with an isolated semioval tip (Iso-Tome) on 25 patients with difficult cannulation: cannulation was successful in 23 patients (92%), five patients developed mild PEP (20%), and one patient had moderate bleeding. However, neither prophylactic pancreatic duct (PD) stents nor rectal NSAIDs were used by the researchers. The same group performed another study using the same Iso-Tome on 59 patients and had a success rate of 86.4%, with four patients developing mild to moderate PEP [9]. Chiu et al. [10] also used a modified design of the isolated papillotome with an angulated tip on 13 hard-to-cannulate cases. They achieved a successful cannulation in all patients with no major complications. Recently, a hybrid Iso-Tome with a smaller and a completely round and insulated tip papillotome (r-ITP) was developed by MTW Endoskopie, Wesel, Germany (Figure 1). This modification might enhance the ease of use and help in reducing the thermal and electrical injury, reducing direct mechanical trauma to the pancreas by controlling the depth of penetration of the needle during manipulation. We conducted a prospective randomized single-center trial to investigate the efficacy and safety of this new round insulated tip papillotome as compared to that of the classic NKS in difficult-to-cannulate ERCP patients.

## 2. Materials and Methods

The study was conducted in the Endoscopy Unit at Bezmialem Vakif University Hospital, a tertiary referral center in Istanbul, Turkey. Between the periods extending from September 2011 to September 2012, all patients with an intact papilla referred for ERCP were screened and invited to participate in the study and then were required to sign an informed consent prior to the procedure. Exclusion criteria included patients with coagulopathy, or those having any anatomical or pathological abnormalities that prevented adequate access, or proper visualization of the papilla. Having a bulging papilla was also considered an exclusion criterion because we believe that classic NKS is safe in these cases due to the created space under the bulge itself and because the bare tip of the needle might be needed in certain cases to create a fistulotomy.

After the screening and exclusion phases, patients with “difficult cannulation” were defined as those requiring more than five minutes to achieve deep biliary cannulation from the first touch of the papilla, while using the regular pull-type sphincterotome with or without guidewire assistance. No more than a total of ten cannulation attempts (defined as a sustained contact between the sphincterotome and the papilla for at least five seconds) were allowed during this time frame. These patients were then randomly assigned to have precut sphincterotomy using either the classic NKS or r-ITP. No crossover between the two groups was permitted. Randomization was performed using a random number generator that was concealed in closed sequentially numbered envelopes. An individual not involved in the study performed the randomization and revealed the allocated intervention when randomization criteria were reached. The endoscopist was blinded to the type of intervention until the envelope was opened. Data were collected regarding the initial indication for the procedure, sex, and age of the patient and presence of associated conditions affecting biliary access like a periampullary diverticulum or previous Billroth II surgery. Success rates and any periprocedural event or complication within the same hospitalization were also recorded. Post-ERCP complications were classified according to consensus guidelines [11]. Primary outcome was the achievement of a successful cannulation. Secondary outcomes were the development of post-ERCP complications, namely, PEP, bleeding, perforation, and death.

The study was approved by the Institutional Review Board of Bezmialem Vakif University, and written informed consent was obtained from all patients. The study was carried out according to Ethical Principles for Medical Research Involving Human Subjects outlined in the Helsinki Declaration.

### 2.1. Statistical Analysis

SPSS version 19 for Windows (SPSS, Chicago, IL, USA) was used for statistical analyses. Descriptive statistical methods (mean, standard deviation, and percentage) as well as Student's *t*-test, Chi-square test, and Fisher's exact tests were used for the evaluation of the study data. Two-tailed *p* values <0.05 were considered statistically significant.

### 2.2. Techniques and Materials

**Figure 2:**
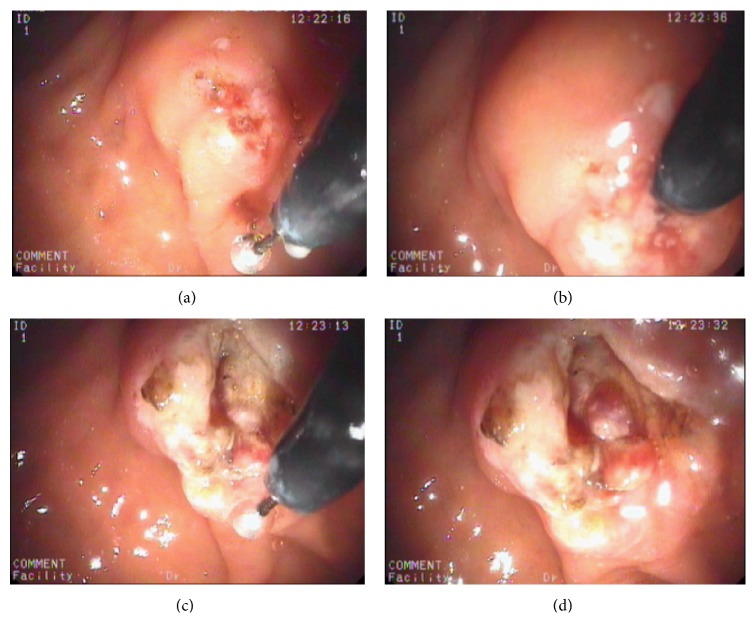
Duodenoscopic findings of precut sphincterotomy with the novel round insulated tip papillotome (r-ITP). (a) r-ITP is introduced in a patient with a prominent ampulla of Vater (AV). (b) r-ITP is placed in the orifice of AV. ((c) and (d)) Bile duct mucosa is exposed after precut sphincterotomy.

All procedures and interventions were performed by a single expert endoscopist (H. Senturk). Procedures were performed under propofol based deep sedation, guided by a dedicated anesthesiology team. A pentax duodenoscope (ED-3680 TK 4.8, PENTAX Tokyo, Japan) was used in all cases, with the patient put in the prone position. An ERBE VIO 300D Electrosurgery Unit (ERBE, Germany) was used with the same settings for both intervention groups using the Endo Cut I mode (effect 2, current set at an output limit of 155 W). It is a monopolar high-frequency electrosurgical procedure, which consists of a two-stage cutting cycle followed by a coagulation cycle. At randomization (after failure of cannulation within 5 minutes/10 attempts frame), the precut papillotomy using the r-ITP (MTW Endoskopie, Wesel, Germany) was performed in a technique similar to that used in the classic freehand deroofing needle-knife papillotomy [8]. After adequate introduction and localization of the r-ITP into the papillary orifice, the operator initiates cutting in two mm increments using the Endo Cut I current while directing the papillotome upwards toward the papillary roof at the 11-12 o'clock position (Figure 2). Exposure of the common bile duct orifice was achieved in most cases, after which cannulation and extension of the sphincterotomy were completed using the conventional pull-type sphincterotomy. A prophylactic PD plastic stent (5 Fr, 5 cm) was placed whenever the PD was inadvertently cannulated. A 100 mg dose of rectal diclofenac suppository was routinely given immediately before the procedure to every patient. Periprocedural intravenous fluid infusion was also administered and tapered to the patient's comorbidities and tolerability. Patients were followed up in the recovery room for six hours after the procedure. Patients with significant abdominal pain were admitted to the hospital for observation and evaluation. A follow-up visit was scheduled within the same week for evaluation of any adverse events.

## 3. Results

**Figure 3:**
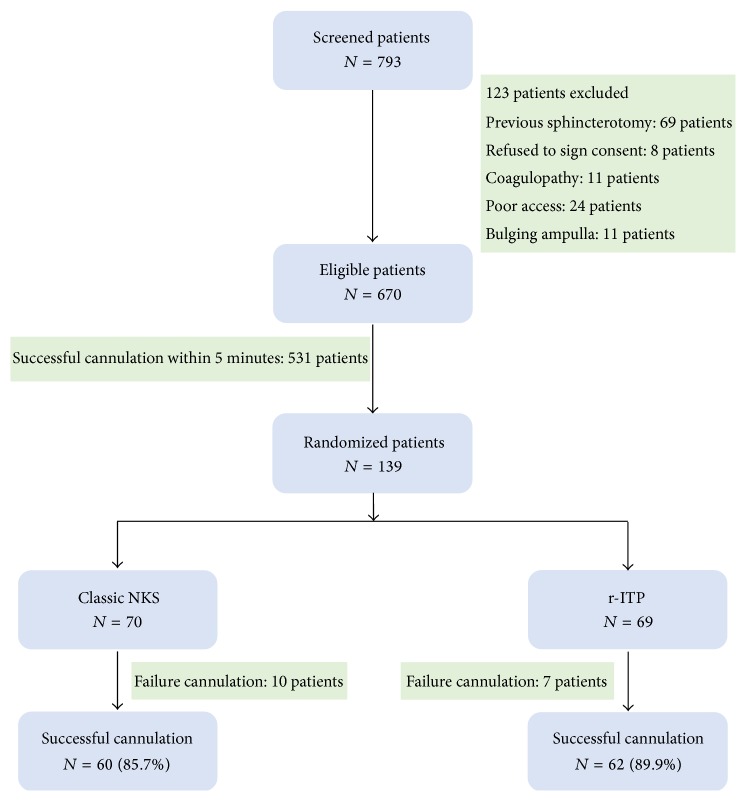
(Flowchart) Distribution of screened, excluded, and randomized patients in the study.

A total of 793 patients referred for ERCP were screened for inclusion and exclusion criteria. Of these, 670 patients were considered eligible, whereas 123 were excluded: 69 were excluded because of having a previous sphincterotomy, eight refused to participate in the study, 11 had a coagulopathy, 24 had anatomical or pathological abnormalities preventing adequate ampullary access, and 11 had a bulging papilla (Figure 3). Successful deep biliary cannulation was achieved in 531 patients (79.3%) within the first five minutes and without exceeding the ten permitted attempts. The remaining 139 patients (20.7%) fulfilled the difficult-to-cannulate criteria and were considered to be eligible for randomization. Seventy patients were randomly assigned to the classic NKS group (40 female/30 male, mean age 60.1 ± 14.4 years, range: 27–86 years), whereas 69 patients were assigned to the r-ITP group (40 women/29 men, mean age 61.1 ± 16.0 years, range: 21–82 years). No difference was noted between the two groups regarding the indication of the procedure. The most common indication was choledocholithiasis (69.8%). Other indications included suspected sphincter of Oddi dysfunction (10%), malignancy (9.4%), and acute pancreatitis (11.5%). On the other hand, a periampullary diverticulum was observed in four patients in the NKS group as compared to three patients in the r-ITP group. Two patients in the NKS group had a history of Billroth II surgery as opposed to three patients in the other group (Table 1).

The primary outcome of successful cannulation was comparable in the two groups (85.7% in the r-ITP group versus 89.9% in the NKS group, *p* = 0.46). The average successful cannulation rate was 87.8%. No statistically significant difference was noted between the two groups. Cannulation failed in seven and ten patients in the r-ITP and the NKS group, respectively. Of these 17 patients, eight were successfully cannulated by a second ERCP attempt on another day. Alternatively, cannulation and drainage were achieved using the rendez-vous technique in three patients, percutaneous transhepatic cholecystostomy in one patient, or surgically in two patients. The remaining three patients refused to perform any other procedure. A prophylactic pancreatic stent was placed in 15 patients (10.8%), seven in the r-ITP group and eight in the NKS group (*p* = 0.81). In total, seven patients had an intervention related adverse event (5%) all of whom were managed conservatively without any major sequel. Three and two patients developed mild to moderate PEP in the NKS and the r-ITP groups, respectively (*p* = 0.67). However, none had severe pancreatitis. One patient in each group had mild bleeding not requiring transfusion. No cases of perforation were encountered (Table 2). No other procedure or anesthesia related major adverse events or mortality was observed.

## 4. Discussion

Achieving successful cannulation without increasing the risk for ERCP related complications remains the ultimate goal in difficult cases. The reported proportion of difficult-to-cannulate patients with conventional methods was variably reported in different studies depending on the definition for failure of cannulation, the experience of the endoscopists, and the contribution of training fellows in the procedure. Failure rates as low as 3.2% [12] and up to 34% [13] have been reported. The success rate with precut sphincterotomy was reported to increase to more than 90% in some studies [14].

NKS is a frequently used modality that was found to significantly improve successful cannulation rates, but it was often preserved as a last resort by most endoscopists for the fear of increased risk of PEP. However, this observed increased risk might be attributed to the repeated cannulation attempts in these difficult cases leading to papillary edema and to repeated inadvertent PD cannulation in many instances, both of which are independently related to increased risk of PEP [15]. A recent meta-analysis [16] of seven RCTs showed a decreased trend for PEP with early precut sphincterotomy (3.9%) as compared to prolonged standard attempts (6.1%) although this failed to reach statistical significance (*p* = 0.07). Another Cochrane meta-analysis, currently available as an abstract [17], showed a significant reduction in the risk of PEP when precut approach was implemented early during the procedure (OR: 0.57, 95% CI: 0.33–0.98). Furthermore, successful cannulation rate was found to be higher with the early intervention protocol (RR: 1.64; 95% CI: 1.28–2.10). Timing for initiation of NKS varied among these trials. Some used it as an initial method [18], while others required 5–20 minutes of failed cannulation attempts. In fact, in order to reduce the risk of PEP, the latest updated ESGE guidelines [19] recommend keeping the number of cannulation attempts as low as possible and suggest that needle-knife fistulotomy should be the preferred precut technique in patients with a bile duct dilated down to the papilla.

Given that all procedures in our study were performed by one highly experienced endoscopist, we adopted the five minutes cutoff trying to particularly minimize PEP risk in this high-risk population. This high threshold might have attributed to the relatively high proportion of the difficult-to-cannulate patients in our population (20.7%). Other concerns with the use of NKS are that the bare end of the needle might slip more easily outside the papillary orifice during manipulation and that it might injure the vessels increasing the risk of bleeding, or injure the wall itself, increasing the perforation risk. These risks might theoretically be reduced while using a round insulated tip, by ensuring a stable needle positioning when in the papilla and having a controlled predictable depth of penetration along with decreasing mechanical as well as electrical injury to the surrounding structures.

To our knowledge, this is the first large RCT to compare two different variants of needle-knife papillotomy. The successful cannulation rate was similar in both groups with an average of 87.8%. Moreover, post-ERCP complications rate was also similar, and r-ITP was not shown to decrease the risk of PEP or bleeding. But interestingly, the overall rate of complications was markedly low: only five patients (3.6%) developed mild-moderate PEP. This low rate as compared to other similar studies, including the pilot study by Park et al. [8] that had a 20% PEP rate, might be attributed to several factors. For instance, a senior endoscopist performed all the procedures and no fellows/trainees were involved in our study. An NSAID suppository was routinely given to all patients, which is known to significantly reduce PEP [20]. Moreover, a prophylactic PD stent was inserted in 10.8% of cases. A recently published systematic review [21], including 18 studies, showed that: pancreatic stent insertion reduces the risk of PEP 13.3%. Recently, Zagalsky et al. [22] reported on the use of early precut sphincterotomy or alternatively pancreatic stent placement for PEP prevention in 100 high-risk patients. PEP rates were similar in the two groups (3.9% versus 4%). No cases of perforation were noted and only two patients had mild controllable bleeding, suggesting that NKS is a generally safe modality when used cautiously.

Our study has several limitations. The endoscopist was not blinded to the modality used due to the visible characteristic needle tip that can be seen during the procedure, which might lead to a preferential bias. We did not calculate the time needed to achieve successful cannulation after randomization, which might affect the risk of PEP. However, the rate of complications in this study was already low. Another limitation was that this was a single-center study, performed by a single expert operator, which makes it difficult to generalize these results to academic or smaller institutions. Furthermore, the endoscopist (H. Senturk) expressed a subjective sense of comfort and safety while using a round insulated tip. The statistical difference between the two groups concerning the efficacy and safety would have been more evident in favor of one of them if used by a less experienced endoscopist.

## 5. Conclusion

In conclusion, early precut sphincterotomy seems to be a very safe modality to enhance cannulation success, especially when used in combination with other prophylactic measures. Round insulated tip papillotome was not shown to be superior to NKS in terms of successful cannulation and post-ERCP complications. However, it remains a simple, safe, and efficacious alternative. Further larger studies in academic centers with less experienced operators might be needed.

## Figures and Tables

**Table 1:** Baseline demographic and clinical characteristics of eligible patients.

	NKS group (*n* = 70)	r-ITP group (*n* = 69)	*p* value
Age, year (mean ± SD)	60.1 ± 14.4	61.1 ± 16.0	0.67
Women, *n* (%)	40 (57.1)	40 (57.9)	0.92
Indications			
Bile duct stone(s), *n* (%)	52 (74.2)	45 (65.2)	0.25
Sphincter of Oddi dysfunction, *n* (%)	6 (8.5)	8 (11.6)	0.56
Acute pancreatitis, *n* (%)	8 (11.4)	8 (11.6)	0.98
Chronic pancreatitis, *n* (%)	4 (5.7)	5 (7.2)	0.72
Malignancy, *n* (%)	7 (10)	6 (8.7)	0.79
Associated conditions			
Periampullary diverticulum, *n* (%)	4 (5.9)	3 (4.3)	0.72
Billroth II operation, *n* (%)	2 (2.9)	3 (4.3)	0.64
Hydatid disease, *n* (%)	0 (0)	1 (1.5)	0.32

**Table 2:** Efficacy and safety of precut papillotomy procedures.

	NKS group (*n* = 70)	r-ITP group (*n* = 69)	*p* value
Successful cannulation, *n* (%)	60 (85.7)	62 (89.9)	0.46
Post-ERCP pancreatitis, *n* (%)	3 (4.9)	2 (2.9)	0.67
Mild bleeding, *n* (%)	1 (1.4)	1 (1.4)	0.99
Prophylactic PD stenting, *n* (%)	8 (11.4)	7 (10.1)	0.81
Perforation, *n* (%)	0 (0)	0 (0)	N/A
Death, *n* (%)	0 (0)	0 (0)	N/A

N/A: not available.
